# Moisture availability in the southwest United States over the last three glacial-interglacial cycles

**DOI:** 10.1126/sciadv.aau1375

**Published:** 2018-10-24

**Authors:** Kathleen A. Wendt, Yuri V. Dublyansky, Gina E. Moseley, R. Lawrence Edwards, Hai Cheng, Christoph Spötl

**Affiliations:** 1 Institute of Geology, University of Innsbruck, Innrain 52, 6020 Innsbruck, Austria.; 2 Department of Earth Sciences, University of Minnesota, 116 Church Street SE, Minneapolis, MN 55455, USA.; 3 Institute of Global Environmental Change, Xi’an Jiaotong University, Xi’an 710049, China.

## Abstract

The projected long-term drying of the southwest (SW) United States in response to climate warming raises a sobering alarm for this already water-limited region, yet the climatic controls on moisture availability over longer time scales remain a topic of debate. Here, we present a 350,000-year record of past water table fluctuations in Devils Hole 2 cave that are driven by variations in recharge amount to the local groundwater flow system. Because of the unprecedented length and precision of our record, we can observe variations in regional moisture availability over the last three glacial-interglacial cycles at a millennial-scale resolution. The timing of past water table rises and falls (>9 m in amplitude) closely coincides with the expansion and reduction of Northern Hemisphere ice volume, which in turn influences the position and intensity of westerly winter storms on orbital time scales. Superimposed on this long-term trend are millennial-scale highstands recorded during the last glaciation that coincide with North Atlantic Heinrich events. Earlier millennial-scale highstands provide the first evidence of multiple short-lived wet periods in the SW United States linked to coeval cooling intervals in the North Atlantic during marine isotope stages 6 and 8. The Devils Hole 2 water table record is currently the longest independently dated paleomoisture record in the SW United States and thus provides a critical testbed to examine the controls on regional moisture availability over larger time scales.

## Teaser

Caves in Death Valley National Park reveal past water table fluctuations in response to 350,000 years of global climate change.

## INTRODUCTION

The Great Basin (GB), located in the southwest (SW) United States, has undergone drastic hydroclimate changes throughout the Quaternary, as illustrated by the expansion and desiccation of large pluvial lakes. Understanding the climatic controls on regional water availability over these longer time scales has become increasingly important, as recent model projections estimate a long-term drying trend in the GB in response to climate forcings (*1*, *2*). The iconic Devils Hole (DH) oxygen isotope record (*3*, *4*) and its recent chronological revisions (*5*) derived from DH and DH2 caves (DH caves) in SW Nevada (LAT: 36.416, LONG: −116.283; ca. 200 m apart) reveal past hydroclimate changes in the GB over an unprecedented time span of 500 thousand years (ka). Yet, until now, much of the attention surrounding the DH record has focused on the proxy of δ^18^O, which is interpreted in the SW United States to reflect changes in temperature, moisture source, and/or seasonality (*3*–*8*). Challenges therefore arise in identifying changes specific to the regional water balance, such as variations in precipitation (P), evapotranspiration (ET), and moisture availability (defined here as an approximate measure of P-ET) using δ^18^O values alone. A more direct proxy of moisture availability includes the dating of ancient shorelines from paleolakes [e.g., (*9*)] and the interpretation of uranium isotopes from pedogenic carbonate and opal deposits (*10*). Yet, these archives are often complicated by fragmented preservation, lack of deposition during nonpluvial conditions, and inherent dating complications such as uncertain reservoir effects. As a result, little is known about past changes in regional moisture availability beyond the last glaciation. An alternative approach to studying moisture availability over longer time scales includes the reconstruction of past water table elevations (a direct proxy for recharge amount) using subaqueous carbonate deposits, which can be precisely dated using uranium-thorium (U-Th) disequilibrium techniques. Reconstructing regional water table elevations in the GB over several glacial-interglacial cycles is critical to examine long-term changes in moisture and test the orbital- to millennial-scale forcings that drive these changes.

**Fig. 1:**
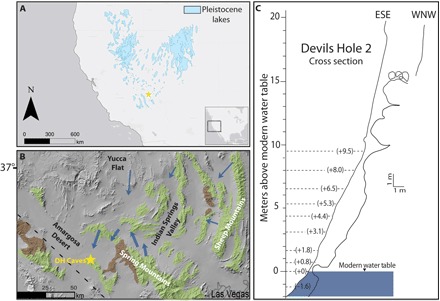
Study site. (**A**) SW United States including the location of GB Pleistocene lakes (https://keck.library.unr.edu/datasets/gbgd.aspx). Yellow star indicates the location of study site. (**B**) Map of groundwater flow system adapted from (*13*, *14*). Blue arrows indicate regional groundwater flow direction. Thicker arrows indicate the major groundwater flow direction of the AMGFS from the Spring Mountains to DH caves. Paleozoic carbonate rock (green) ridges broadly reflect the spatial extent of a large, connected lower carbonate rock aquifer. Lower Cambrian siliciclastic rock (brown) ridges indicate the location of local aquitards. Yellow star indicates the location of DH caves (located ca. 200 m apart). (**C**) Cross section of DH2 cave. Dashed lines indicate the location of cores drilled into the hanging cave wall. ESE, east-southeast; WNW, west-northwest.

Here, we present a high-precision record of moisture availability in the GB over the last three glacial-interglacial cycles as interpreted through fluctuations in water table height recorded in DH2 cave. DH caves intersect the Ash Meadows groundwater flow system (AMGFS) located within a large (ca. 12,000 km^2^) Paleozoic carbonate basin (Fig. 1). Pioneering work by Szabo *et al*. (*11*) demonstrated that AMGFS water table fluctuations recorded in DH cave are driven by changes in recharge amount over the past 100 ka and thus reflect variations in regional moisture availability through time. Using newly collected samples from DH2 cave, we have refined and expanded this record to span the past 350 ka.

Recharge to the AMGFS is primarily sourced from the Spring Mountains (~60 km southeast from DH caves), with average annual precipitation exceeding 600 mm at the crest [3600 m above sea level (a.s.l.)] (*12*). Additional minor groundwater contributions are estimated to be sourced from eastern and northeastern basins (*13*, *14*). Approximately 90% of groundwater recharge at Spring Mountains is sourced from snowpack melt (*12*). Regional snowfall is principally derived from winter storms that move east-southeast along the Pacific storm track (*12*, *14*), a narrow zone of extratropical cyclones transporting North Pacific– and Central Pacific–sourced moisture inland. The remaining ~10% of groundwater recharge is derived from summer precipitation sourced from a northerly expansion of the North American monsoon and is considered negligible with regard to total moisture contribution (*12*). Large shifts in GB moisture availability on glacial-interglacial time scales are largely attributed to variations in the position and intensity of the Pacific storm track (*9*, *15*, *16*), although arguments for northward perturbations of tropical Pacific-sourced air masses have been proposed (*17*).

Because of the extensional tectonic regime dating back to the mid-Tertiary, the AMGFS groundwater flows through a network of fractures that allow for the transportation of large volumes of water with minimal influence from surface topography (*13*, *14*). Both caves developed within a set of SW-striking fractures in the groundwater discharge center of the AMGFS. Recent surveying by the authors shows identical water table elevations within measurement uncertainties of 8 cm. Because of the long flow path (>100 km) and long groundwater transit times (<2000 years) (*4*), the groundwater that passes through both caves is supersaturated (saturation index = 0.2) with respect to calcium carbonate (*18*) and has been continuously depositing secondary calcite on the submerged cave walls for more than 500 ka (*3*). Petrographic and morphological differences between calcite precipitated below (known as mammillary) and at the water table (known as folia; fig. S7) in DH caves provide a natural hydrograph of past variations in water table elevation (*11*). Mammillary calcite depositing subaqueously features a dense (porosity, <1%) fabric with average growth rates of about 1 mm ka^−1^ (*4*, *5*). In contrast, calcite formed at the water table (folia) creates shelf-like structures characterized as a variably porous (<1 to 20%), bright white, faster depositing speleothem (*11*). In this study, we date the onset and cessation of mammillary calcite deposition at various elevations within DH2 cave to reconstruct the height of the local water table over the past 350 ka.

## RESULTS

Ten cores were drilled horizontally at incremental elevations between −1.6 and +9.5 m relative to the modern water table (r.m.w.t.) along the hanging wall of DH2. We macroscopically identified petrographic boundaries along the growth axis of each core (see Materials and methods). Changes from folia calcite (deposited at the water table) to mammillary calcite (deposited below the water table) indicate periods of rising water table, whereas changes from mammillary to folia calcite indicate periods of decreasing water table (fig. S1). We determined the timing, elevation, and direction of changes in the water table over the past 350 ka from an extensive suite of 116 U-Th dates subsampled from mammillary calcite adjacent to petrographic boundaries [table S1; note that folia calcite cannot be reliably dated using U-Th techniques (*5*)]. Wherever possible, the age of petrographic boundaries was determined with three separate U-Th dates to (i) demonstrate stratigraphic consistency and (ii) model ages directly at the boundary using OxCal P sequence (fig. S2) (*19*). In total, we determined the ages of 72 petrographic boundaries using U-Th techniques (table S2). Cores from +9.5, +8, +6.5, +5.3, and +4.4 m r.m.w.t. contain some intervals of mammillary calcite that are too thin (≤0.5 mm) to be dated (fig. S1). These thin mammillary layers, however, unambiguously indicate that the water table was higher during deposition of these intervals. We therefore estimate the age of these thin layers by stratigraphically aligning them using well-dated evidence from cores below and above the respective elevations (see Materials and methods). Figure S3 shows all 116 U-Th dates, 72 petrographic boundaries, and 25 estimated ages of thin mammillary calcite layers. The lowest core sampled at −1.6 m r.m.w.t. is composed entirely of mammillary calcite, indicating that the water table in DH caves reached no lower than −1.6 m r.m.w.t. over a minimum of the last 350 ka.

**Fig. 2:**
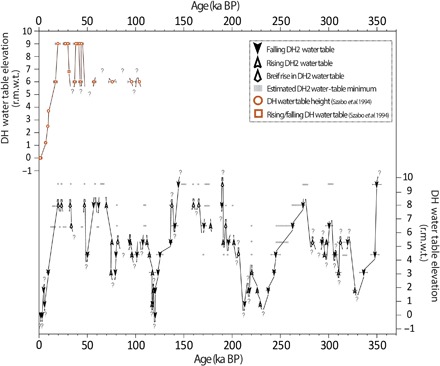
DH caves water table records. DH record [orange; (*11*)] and DH2 record (black; this study) plotted r.m.w.t. with 2σ uncertainties. Modern water table elevations are identical in both caves (see main text). Arrows represent inferred water table direction. Dashed horizontal bars represent estimated water table minimums, as determined by the presence of thin mammillary calcite layers (see main text). Question marks denote uncertainties in water table minima/maxima. BP, before present (1950 A.D.).

The DH2 water table record reveals orbital-scale fluctuations with a range of nearly 10 m over the past 350 ka, as well as many lower-amplitude millennial-scale events (Fig. 2). The portion of our record with the highest resolution spans the past 100 ka, during which 23 petrographic boundaries dated with the chronological precision of up to 3‰ capture 12 millennial-scale oscillations in water table elevation. These results demonstrate remarkable similarity to the DH water table record (*11*), thus providing an important test of reproducibility and the high connectivity of the AMGFS (Fig. 2). In addition to improving the resolution of the record over the last glacial cycle, our data also provide new insights into water table fluctuations over the penultimate and antepenultimate glacial cycles, back to 350 ka.

Water table highstands (above +5.5 m r.m.w.t.) at 350 (±5) ka, 320 to 262 (±3) ka, 201.8 to 136.3 (±0.9) ka, and 111.8 to 19.85 (±0.4) ka broadly correspond to marine isotope stage (MIS) 10, MIS 9d-8, MIS 7a-6, and MIS 5d-2, respectively, although superimposed on these broad-scale highstands were many smaller-scale oscillations. Water table lowstands (lower than +1.8 r.m.w.t.) are centered at 327 (±4) ka (MIS 9e), 237 (±1) ka (MIS 7e), 213 (±1) ka (MIS 7c), 120 (±0.5) ka (MIS 5e), and 2.76 (±0.03) ka (MIS 1).

## DISCUSSION

Processes that may drive water table fluctuations in the AMGFS over the past 350 ka include changes in recharge amount, the inflow/outflow into/from neighboring basins, and regional tectonism (see the “*Processes impacting water-table elevation*” section in the supplementary materials). The synchronicity of observed highstands and lowstands to the timing of the last three glacial-interglacial cycles strongly argues against regional tectonism and varying connections to adjacent basins as primary controlling factors. Instead, our data support the mechanism previously proposed by Szabo *et al*. (*11*) that AMGFS water table fluctuations are primarily driven by variations in recharge amount, such that, during glacial (interglacial) periods, a wetter (drier) climate would result in increased (decreased) recharge amount to the AMGFS, thereby raising (lowering) the water table. Periods of high water table elevations are consistent with wet periods interpreted from paleorecords located in the surrounding AMGFS region, including spring deposits in Las Vegas valley (*20*), growth of speleothems in the southern Spring Mountains (*21*), and the evaluation of plant macrofossil assemblages from packrat middens located within the Nevada Test Site, which indicate an increase in local precipitation of up to 40% relative to today during the latter half of the last glaciation (*22*). Across the wider GB region, periods of high water table elevations are consistent with expansions of Sierra Nevada glaciers (*23*), increased infiltration recorded in pedogenic carbonate and opal deposits (*10*), and the filling of numerous GB lakes, including the large closed-basin lakes of Bonneville, Lahontan, and Manly (fig. S4). We therefore interpret DH2 water table fluctuations to reflect changes in local moisture availability and provide insight into the paleohydroclimate of the wider SW United States region.

**Fig. 3:**
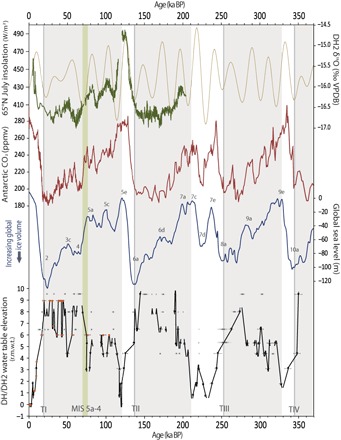
Three hundred fifty thousand years of DH caves water table fluctuations in comparison with local δ^18^O, solar insolation, and global ice volume. NHSI (gold) (*24*); DH2 cave δ^18^O (green) relative to Vienna Pee Dee Belemnite (VPDB) (*5*). Atmospheric *P*co_2_ (red) recorded in European Project for Ice Coring in Antarctica Dome C (*25*). Global sea level (*26*) with associated MISs labeled. Water table fluctuations recorded in DH cave [orange; (*11*)] combined with water table fluctuations recorded in DH2 cave (black; this study) including 2σ uncertainties. DH caves water table fluctuations plotted r.m.w.t. Shaded regions represent glacial periods. Green bar represents the MIS 5a-4 boundary. TI to TIV, terminations I to IV; ppmv, parts per million by volume.

The timing of large (frequently >8 m) decreases in water table elevations starting at 19.85 (±0.04) ka, 137.6 (±0.5) ka, 221 (±2) ka, and 350 (±5) ka broadly coincides within dating uncertainties to periods of increasing 65°N July insolation [Northern Hemisphere summer insolation (NHSI)] (*24*), increasing atmospheric CO_2_ concentrations (*25*), and sea level rise (*26*) associated with glacial terminations IV, II, and I (Fig. 3). An exception is termination III in which decreasing water table elevations commenced at 273 (±2) ka (although the possibility of brief highstands after this time cannot be excluded) and may be linked to the onset of rising atmospheric CO_2_ and NHSI at this time. In total, the DH water table record reveals that large-scale shifts in GB moisture availability during glacial-interglacial transitions are driven by mechanisms ultimately tied to orbital forcings.

Over the past 350 ka, water table highstands (lowstands) recorded in DH caves coincide with decreased (increased) DH2 δ^18^O values (*5*), decreased (increased) atmospheric *P*co_2_ (partial pressure of CO_2_) (*25*), and increased (decreased) global ice volume, as indicated by decreased (increased) global sea level (Fig. 3) (*26*). The synchronicity of these distant records is likely tied to variations in Northern Hemisphere ice volume, including the thickness and extent of the North American continental ice sheets and neighboring sea ice. Although the underlying processes remain an ongoing debate (*17*, *27*), model-proxy comparisons suggest that an increase in NHSI-forced Northern Hemisphere ice volume (further enhanced by decreasing *P*co_2_) contributed to a steepening of the meridional sea surface temperature gradient across the eastern North Pacific, which deepened the Aleutian low and strengthened the subtropical jet (*15*, *16*, *28*). Combined, these processes represent key variables in determining the strength, frequency, and direction of winter storms along the Pacific storm track (*29*), resulting in increased moisture transport to the North American SW (*15*, *16*, *28*).

**Fig. 4:**
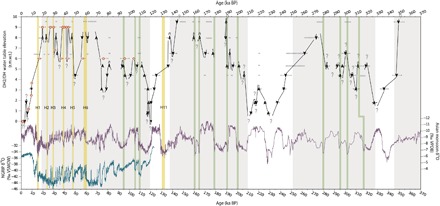
Millennial-scale events recorded in DH caves over the last three glacial periods. DH [orange; (*11*)] and DH2 (black; this study) plotted r.m.w.t. with 2σ uncertainties. Asian monsoon speleothem δ^18^O values (purple) record the relative intensity of the East Asian monsoon, which is sensitive to North Atlantic variability (*36*). North Greenland Ice Core Project (NGRIP) ice core record (blue) record relative North Greenland temperatures (*30*). Large shaded regions represent glacial periods. Yellow bars indicate the timing of Heinrich events (H) 1 to 6 and 11 [determined by (*35*)]. Green bars indicate North Atlantic cool periods, including those inferred from low monsoon intensity during MIS 6 and MIS 8. VSMOW, Vienna Standard Mean Ocean Water.

Focusing on the portion of our record with the greatest chronological control, the last glacial period is characterized by two major rises in the water table leading to broad highstand intervals at MIS 5d-a and MIS 4-2 that are interspersed with millennial-scale fluctuations. The lowstand of the last interglacial ended sometime between 120.4 and 118.3 (±1.2) ka and coincides with the initial expansion of global ice volume and decreasing Northern Hemisphere high-latitude temperatures (Figs. 3 and 4) (*30*). A second major rise occurred during the transition between MIS 5a and 4, during which the water table elevation rose a minimum of 6.3 m (<+3.1 to >+9.5 m r.m.w.t.) starting between 78.0 and 74.4 ka. The MIS 5a-4 boundary rise in water table elevation coincides with the initial filling of large GB lakes (e.g., Cutler dam cycle of Lake Bonneville; fig. S4). Curiously, the GB lakes do not appear to have responded during the pluvial phases of MIS 5d-a despite increases in moisture availability recorded in DH2 cave. This may be due to a lack of observational data, but more likely, a threshold in moisture availability was needed to sustain the large lakes. Our data support this hypothesis by indicating sustained higher water table elevations for longer periods of time during MIS 4-2 compared to MIS 5d-a. The series of events leading up to MIS 4 suggests that an increase in Northern Hemisphere ice volume contributed to a southward displacement and/or intensification of the Pacific storm track that, when coupled with cooler surface temperatures (*5*), resulted in increased infiltration across the GB. A rapid rise in water table elevations during the MIS 5a-4 transition may be due to the intensification of these processes, including a rapid expansion of North American ice sheets at this time (*31*), which ultimately split midlatitude westerlies (*15*) and drove winter storms further southward. Overall, our record supports the hypothesis that Northern Hemisphere ice volume directly influenced the North Pacific meridional temperature gradient and position of stationary pressure centers, which in turn acted as primary drivers of the Pacific storm track position and intensity on orbital time scales.

Throughout MIS 4-3, we observe millennial-scale water table fluctuations reaching more than 3.5 m in elevation difference (Fig. 4). We record water table highstands in DH caves at 61.7 (±0.1) ka, 47.0 (±0.1) ka, 42 (±2) ka, 30 (±2) ka, and 23.2 (±0.1) ka, which broadly correlate in time with wet periods recorded in SW United States speleothems [e.g., (*6*, *7*, *21*, *32*)] and paleolakes [e.g., (*9*, *33*, *34*)], and correspond in time to North Atlantic Heinrich events 6 to 2 (*35*). The timing of recorded water table highstands supports a previously proposed indirect yet rapid link between temperature variations over the North Atlantic and winter precipitation over the SW United States (*6*–*9*, *21*, *32*–*34*). Following the Last Glacial Maximum (water table highstand more than +8 m r.m.w.t. at 19.85 ± 0.04 ka), thin mammillary calcite layers are identifiable in the DH2 core located at +8 m r.m.w.t. These brief highstands may correspond to the GB wet periods associated with Heinrich event 1 and/or the Younger Dryas, which are recorded in several northern GB paleolakes (*33*). Similarly, thin layers of mammillary calcite are identifiable following the MIS 6 glacial maximum (water table highstand more than +8 m r.m.w.t. at 137.6 ± 0.5 ka) and may correspond to the GB wet period associated with Heinrich event 11 (*8*). The absence of prominent highstands associated with postdeglaciation Heinrich events is attributed to a continual decrease in SW GB precipitation following the glacial maxima, as supported by Nevada pedogenic carbonate and opal deposits (*10*), due to a gradual northward recovery of the Pacific storm track. Paleolake networks suggest that the mean position of the Pacific storm track reached approximately 41°N before Heinrich event 1 (*33*). Paleolake highstands that coincide with Heinrich event 1 are predominately located in the northern GB [e.g., (*9*, *34*)], indicating that augmented westerly moisture flow associated with Heinrich event 1 was largely diverted to the northern GB [see (*33*)]. Lower precipitation rates in the SW GB relative to previous Heinrich events could inhibit a prolonged water table highstand during Heinrich event 1 and 11, while still registering in proxies more sensitive to changes in the SW GB water balance and surface temperatures [e.g., (*21*)].

Earlier millennial-scale highstands during the last glaciation correspond within age uncertainties to Greenland Stadials (GS) 25, 24, and 23 (note a gap in the DH record during GS 22). A steady rise in water table elevations throughout the MIS 5a-4 transition likely overshadowed any millennial-scale variability during GS 21-20, thereby further underscoring ice volume as a dominate forcing in GB moisture availability. Our record provides the first evidence for millennial-scale variations in GB moisture availability before the last glaciation (Fig. 4). To examine the timing of MIS 6 and MIS 8 water table highstands in relation to millennial-scale North Atlantic variability, we compare the DH water table fluctuations to the well-dated Asian monsoon record (*36*). The Asian monsoon intensity is highly sensitive to North Atlantic variability, as demonstrated by the close correlation to North Greenland temperatures (*30*) during the last glaciation. MIS 6 water table highstands (above +5.3 m r.m.w.t.) at 201.8 (±0.9) ka, 189.95 (±0.7) ka, 177.7 (±0.9) ka, 165.3(±0.7) ka, and 159.9 (±0.7) ka coincide within age uncertainties to periods of low monsoon intensity (indicating cooler North Atlantic conditions). Water table highstands (above +5.3 m r.m.w.t.) at 312 (±3) ka, between 307 and 301 (±5) ka, at 296 (±2) ka, and between 283 and 273 (±2) ka coincide within age uncertainties to MIS 8 low monsoon intensity intervals. Overall, the DH2 water table record provides the first evidence linking millennial-scale North Atlantic cooling intervals to multiple GB wet periods during MIS 6 and MIS 8, likely facilitated through similar mechanisms that prevailed during MIS 4-3.

In addition to time series comparisons, the frequency and duration of water table highstands recorded in DH caves provide new insights into GB pluvial conditions associated with the largely understudied MIS 8 and MIS 6. Concentrating on the highest collected core at +9.5 m r.m.w.t., we find a number of intervals of mammillary calcite, indicating times when the water table was in excess of this elevation. However, only three mammillary intervals are thick enough for reliable U-Th dating, yielding ages of 144.3 (±0.6) ka, 190.0 (±0.7) ka, and 350.0 (±4.7) ka (fig. S5 and table S1). Following subaqueous deposition at 144.3 (±0.6) ka, four mammillary calcite layers are observed that are too thin to date with precision (Fig. 2). Based on stratigraphy and age constraints from other cores, these layers were likely deposited during the last glacial period. In contrast, more than 10 thin intervals of mammillary calcite were deposited during the penultimate glacial MIS 6 between 190.0 and 144.3 (±0.7) ka, while no mammillary calcite in excess of 0.5 mm thick was deposited during MIS 8. Given the higher frequency and longer durations with which the water table reached in excess of +9.5 m r.m.w.t. during MIS 6 compared to MIS 2 and even MIS 8, we infer that MIS 6 was the wettest of these glacial periods. This is supported by radiometrically dated paleoshorelines from Lake Manly and Lake Owens (fig. S4), located a respective ~50 and ~150 km west of DH caves, where higher lake levels have been documented during MIS 6 as compared to the last glacial period (*37*, *38*). Similarly, dated moraines in the eastern Sierra Nevada Mountains (*23*) indicate more extensive glacial advances during the MIS 6 glacial maximum relative to MIS 2 (fig. S4). Combined, paleolake and alpine glacier evidence points to more humid conditions in the SW GB during MIS 6 in comparison to the last glacial period. Higher averaged DH water table elevations sustained throughout MIS 6 support this hypothesis and provide the first evidence for more humid MIS 6 conditions relative to MIS 8. Increased moisture availability throughout MIS 6 points to differing atmospheric circulation patterns that influenced the SW during this glacial interval, perhaps forced by the position and extent of the MIS 6 Northern Hemisphere continental ice sheet and sea ice.

A firm understanding of the controls on regional water availability in the GB is critical in predicting the future of this climatically sensitive region. Long-term records of moisture availability in the GB provide a testbed in which possible controls can be examined. Water table fluctuations recorded in DH caves indicate a link between GB moisture availability, global ice volume, and atmospheric CO_2_ concentrations over the past 350 ka (Fig. 3), thereby providing robust paleoevidence in support of long-term projections (*1*, *2*), suggesting continual drying of the GB in response to future climate change.

## MATERIALS AND METHODS

### Core sampling

All sampling for this study was conducted in DH2 under research permit numbers DEVA-2010-SCI-0004 and DEVA-2015-SCI-0006 issued by the Death Valley National Park. Ten cores were drilled between −1.6 m below and +9.5 m above the modern water table (719 m a.s.l.).

### U-Th dating measurements

Identification of the precise petrographic boundaries (based on distinct color, fabric, and porosity differences between folia and mammillary calcite) was performed using light microscopy. Evidence reported in (*5*) suggests that folia layers cannot be reliably dated, likely because of uranium leaching commonly occurring in high-porosity samples. Instead, all U-Th subsamples were drilled in the mammillary calcite immediately above and below each folia layer (fig. S1). U-Th samples were hand drilled using carbide-tipped drill bits 0.3 to 0.4 mm in diameter. To account for the slight offset between the location of the U-Th sample and the precise location of the mammillary/folia boundary, respective growth rates (range, 0.7 to 1.0 mm ka^−1^) were used to calculate the number of years of growth in the interval between the nearest U-Th age and the boundary. Where the number of years in this offset exceeded the dating uncertainties of the nearest U-Th age, three consecutive U-Th samples were measured in close succession (fig. S1). This enabled modeling of the boundary age (fig. S2) and also provided confidence in the dating by demonstrating stratigraphic consistency close to major fabric changes. In addition, 13 of 72 boundaries included a calcite layer that was approximately 1 mm in width before folia deposition resumed. We interpreted these thin layers as a brief increase in the water table. A single date was measured in these cases, and the reported brief increase in water table elevation was centered on the measured date (fig. S1).

Powdered sample sizes ranged between 30 and 50 mg of calcite. U-Th dating was performed at the University of Minnesota. Samples were digested in acid and spiked with a mixed ^233^U-^236^U-^229^Th spike similar to that described in (*39*). Spiked samples were fumed with concentrated HClO_4,_ coprecipitated with iron, centrifuged, and loaded into anion exchange columns. Separate uranium and thorium liquid extracts were measured on a Thermo Neptune multicollector inductively coupled plasma mass spectrometer (MC-ICP-MS) following the methods described in (*40*). Chemical blanks were measured with each set of 10 to 15 samples and were negligible (<50 ag of ^230^Th, <100 ag of ^234^U, and <0.5 pg of ^232^Th and ^238^U per sample). Full U-Th results are listed in table S1.

### Extrapolation

Extrapolated boundary ages were calculated using the OxCal P sequence age model (fig. S2) (*19*). In total, 31 of 72 boundary ages were modeled and yielded dating uncertainties of less than 4900 years (2σ), which are larger yet comparable to U-Th ages (table S2).

### Interpretation of thin mammillary calcite layers

Mammillary calcite (subaqueously deposited) intervals (≤0.5 mm in width) were identified in cores H (+9.5 m), I (+8 m), N (+6.5 m), J (+5.3), and G (+4.4). Because of spatial limitations associated with hand-held drilling techniques, these intervals could not be confidently dated without risking the incorporation of folia material. Instead, identified intervals with thin mammillary calcite layers are bracketed in age by (i) the following/previous dated boundaries along the same core and (ii) the presence of U-Th–dated folia boundaries that were deposited lower in vertical height, suggesting that, at the time of “lower” folia deposition, mammillary calcite layers could not be deposited above this height. For example, thin mammillary calcite layers were identified in core N (+6.5 m) between 262 and 193 ka. These layers are further constrained in age by evidence for a progressively decreasing water table level from +4.4 m at 245 ka. The water table remained below +4.4 m until 220.5 ka, when a folia-to-mammillary calcite boundary suggests that the water table rose above +3.1 m. It is possible that the water table could have risen to +6.5 m and deposited one or more thin mammillary calcite layers at this time (corresponding to MIS 7d). The end of mammillary calcite deposition is constrained by evidence for a decreasing water table at +1.8 dated to 217.4 ka. As shown in Fig. 2, a striped gray bar at +6.5 m between 220.5 and 217.4 ka represents this possible period of mammillary calcite deposition. Although independently constrained in time, we emphasize that the timing of these thin mammillary calcite deposits are estimations.

## Supplementary Material

http://advances.sciencemag.org/cgi/content/full/4/10/eaau1375/DC1

## SUPPLEMENTARY MATERIALS

Supplementary material for this article is available at http://advances.sciencemag.org/cgi/content/full/4/10/eaau1375/DC1 Supplementary background information Fig. S1. U-Th age sampling and extrapolation diagram. Fig. S2. Example of OxCal modeled petrographic boundary. Fig. S3. DH2 water table record including nonextrapolated U-Th ages. Fig. S4. Past regional moisture availability recorded in the GB. Fig. S5. Real-color scanned image of core H collected at +9.5 m r.m.w.t. Fig. S6. Real-color scanned image of all DH2 cores included in this study. Fig. S7. Photo of folia and mammillary calcite in DH cave. Table S1. U-Th dating results. Table S2. Age and location of petrographic boundaries. References (*41*–*45*)
