# Supplementary material for: Moisture availability in the southwest United States over the last three glacial-interglacial cycles
Source: Sci Adv. 2018 Oct 24;4(10):eaau1375. doi: 10.1126/sciadv.aau1375 (PMC6200359; doi:10.1126/sciadv.aau1375)
Supplement: http://advances.sciencemag.org/cgi/content/full/4/10/eaau1375/DC1 [file supp_4_10_eaau1375__index.html]

Science Advances | Science Advances

## Supplementary Materials

**This PDF file includes:**

- Supplementary background information
- Fig. S1. U-Th age sampling and extrapolation diagram.
- Fig. S2. Example of OxCal modeled petrographic boundary.
- Fig. S3. DH2 water table record including nonextrapolated U-Th ages.
- Fig. S4. Past regional moisture availability recorded in the GB.
- Fig. S5. Real-color scanned image of core H collected at +9.5 m r.m.w.t.
- Fig. S6. Real-color scanned image of all DH2 cores included in this study.
- Fig. S7. Photo of folia and mammillary calcite in DH cave.
- Table S1. U-Th dating results.
- Table S2. Age and location of petrographic boundaries.
- References (*41*–*45*)

Download PDF

**Files in this Data Supplement:**

- Adobe PDF - aau1375\_SM.pdf
