# Supplementary material for: Moisture availability in the southwest United States over the last three glacial-interglacial cycles
Source: Sci Adv. 2018 Oct 24;4(10):eaau1375. doi: 10.1126/sciadv.aau1375 (PMC6200359; doi:10.1126/sciadv.aau1375)
Supplement: http://advances.sciencemag.org/cgi/content/full/4/10/eaau1375/DC1 [file aau1375_SM.pdf]

Science Advances
AAAS

advances.sciencemag.org/cgi/content/full/4/10/eaau1375/DC1

Supplementary Materials for

Moisture availability in the southwest United States over the last three glacial-interglacial cycles

Kathleen A. Wendt*, Yuri V. Dublyansky, Gina E. Moseley, R. Lawrence Edwards, Hai Cheng, Christoph Spötl

*Corresponding author. Email: kathleen.wendt@uibk.ac.at

Published 24 October 2018, *Sci. Adv.* **4**, eaau1375 (2018)
DOI: 10.1126/sciadv.aau1375

This PDF file includes:

Supplementary background information
Fig. S1. U-Th age sampling and extrapolation diagram.
Fig. S2. Example of OxCal modeled petrographic boundary.
Fig. S3. DH2 water table record including nonextrapolated U-Th ages.
Fig. S4. Past regional moisture availability recorded in the GB.
Fig. S5. Real-color scanned image of core H collected at +9.5 m r.m.w.t.
Fig. S6. Real-color scanned image of all DH2 cores included in this study.
Fig. S7. Photo of folia and mammillary calcite in DH cave.
Table S1. U-Th dating results.
Table S2. Age and location of petrographic boundaries.
References (*41–45*)

Supplementary background information

Ash Meadows Groundwater Flow System

The Ash Meadows groundwater basin is formed by Paleozoic carbonate rock hosting a regional groundwater flow system. Hydrogeological conditions of the Ash Meadows groundwater basin are estimated to have remained constant over the last 2-3 million years (*13*). Groundwater flows through carbonate aquifer along a network of NE-SW orientated extensional fractures and is little affected by surface topography. Due to the high transmissivity of this aquifer, (a) water-table heights are estimated to rapidly respond to variations in recharge amount and (b) fluctuations of the water-table recorded in the Devils Hole caves are representative of the system as a whole. AMGFS groundwater discharge occurs primarily along the Ash Meadows spring line in the eastern Amargosa Desert approximately 1 km (minimum distance) from DH caves.

Devils Hole and Devils Hole 2 caves

The DH caves are tectonic (extensional) in origin. Devils Hole 2 cave is located c. 200 m NNE (upstream) from Devils Hole proper. The hydraulic gradient is low (0.06 m km^{-1}), and the travel time estimated at <2000 years (*4*). The groundwater transit time between Devils Hole proper and Devils Hole 2 is 5 years (*5*). The fissures in both caves is mostly less than 2 m wide and extends to depths of over 150 m; however, the water table at the Devils Hole proper site is open to the surface c. 15 m above, while the Devils Hole 2 water table is located c. 40 m below the surface. Today, folia exists in Devils Hole 2 cave between +20 m to -0.8m r.m.w.t. Mammillary and folia calcite is hypothesized to have ceased deposition after the Devils Hole 2 roof collapse at 4 ka (*5*). Thus, the vertical extent of folia represents the minimum position of the water table at that time.

Modern water-table levels

Modern water-table levels at Devils Hole caves are approximately 30 cm below pre-pumping levels that began in the early half of 1900s (*personal comm. with K. Wilson of the Death Valley National Park Services*). Recent surveying by the authors shows identical water-table elevations between both caves within measurement uncertainties of 8 cm.

Processes impacting water table elevation

Using uranium-uranium dating techniques (with estimated initial $\delta^{234}U$ values) on three surface calcite veins located in the Ash Meadows region, Winograd and Szabo (1986) calculated an average rate of water-table lowering to less than -0.1m/1000 yr over the past 1 million years, attributed to tectonic uplift

at 0.2 to 0.6 m/ka (*41*). A unilateral lowering of the AMGFS water table was later called into question when the Devils Hole water-table record (*11*) revealed increases in water-table elevations in association with pluvial periods, indicating that climate variations (not uplift) are the primary driver of water-table changes over the last 100 ka. Although an underlying process of long-term lowering cannot be excluded, the Devils Hole water-table record introduced the possibility that the Ash Meadow surface veins sampled by Winograd and Szabo (1986) may have been deposited during similar pluvial periods, thereby representing a maxima water-table height during that time. Assuming this to be true, Devils Hole water-table maxima (this study; *11*) compared to Ash Meadow surface veins (*41*) suggest underlying water-table lowering rates of less than -0.002 m/ka over the last 500 ka, and is therefore considered negligible to 350 ka record presented here. Additional processes that could potentially trigger changes in the water-table elevations in DH caves include:

a) regional tectonism
b) secondary calcite deposition/erosion within the groundwater system or neighboring springs
c) variations in/outflow from/into adjacent groundwater flow systems
d) changes in the recharge amount

Regional tectonic extension may influence the local water-table elevation by (a) facilitating a partial release of the 50-m head differential between DH caves and neighboring Ash Meadows springs or (a) increasing the transmissivity of the aquifer. Other regional tectonic deformations, such as tilting, warping, or vertical offsets, may directly alter relative water-table elevations or indirectly influence elevations through changes in the transmissivity. We argue against regional tectonism as the primary driver of AMGFS water-table elevations over the last 350 ka, as they would likely result in random and unilateral displacement in the water table and therefore cannot explain the close synchronicity of water-table oscillations with the last four glacial-interglacial cycles. Secondary calcite deposition may decrease permeability within the aquifer or restrict flow in neighboring springs (and conversely for calcite erosion). Yet, similar to regional tectonism, one would expect these changes in transmissivity and/or flow restriction to induce unilateral changes in the water-table elevation and cannot sufficiently explain the glacial-interglacial and millennial-scale changes observed in our record. Varying inflow from neighboring aquifers remains a possibility, but is likely linked to variations in recharge amount to these neighboring aquifers resulting in an overflow to AMGFS. Variations of groundwater outflow into neighboring aquifers is unlikely unless water-table elevations surpass a major trough in the potentiometric surface approximately 3 km southwest of DH caves (*13*). All factors considered, we conclude that AMGFS water-table fluctuations are primarily driven by variations in climate over the last 350 ka.

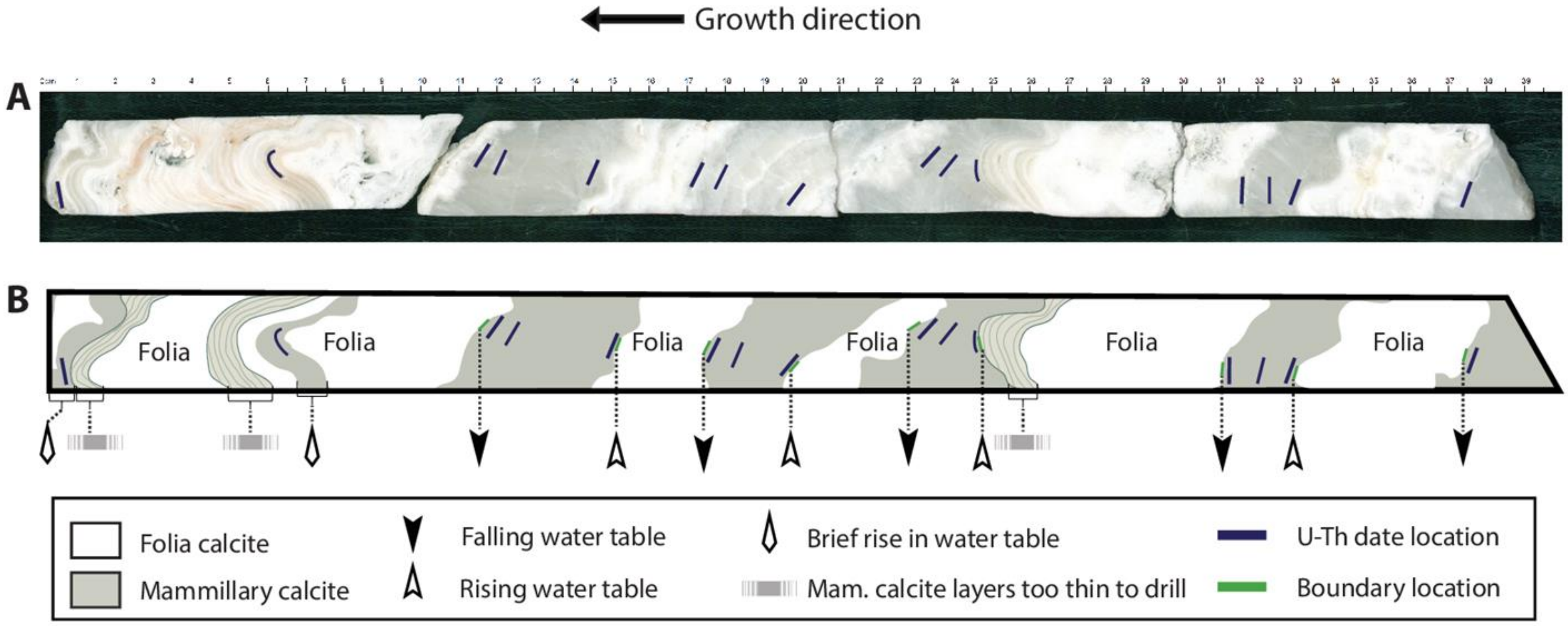

Fig. S1. U-Th age sampling and extrapolation diagram. A: Image of Devils Hole 2 core drilled from the hanging wall at +9.5m r.m.w.t. Folia (annotated white material) reveals the height of the water table (+9.5m) at the time of its deposition. The age of folia deposits are constrained by drilling one to three U/Th ages (blue lines) above/below each folia layer and extrapolating (brown arrow) to the precise onset/termination of folia deposition (i.e. folia boundary). For more information on age extrapolation, please refer to the methods section. The onset of folia growth indicates a decreasing water table (black triangle), the termination of folia growth (folia calcite to mammillary calcite) indicates an increasing water table (white triangle), and a thin mammillary calcite layer (between approximately 3 to 0.5mm) bracketed by folia deposits indicates brief rises in the water table (white diamonds). The location of mammillary calcite layers too thin to U-Th date (<0.5mm) are represented by faded grey rectangles. The age of calcite layers too thin to date is constrained by dated deposits located above and below its elevation.

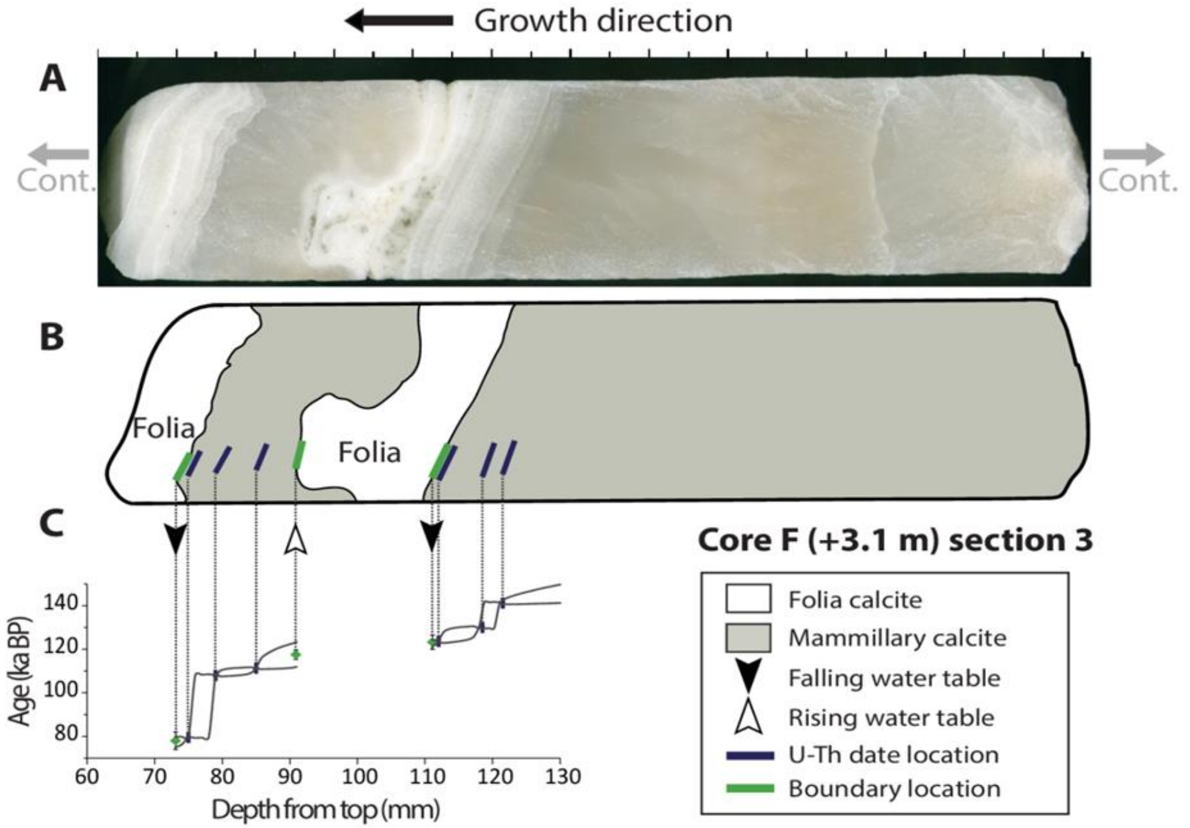

Fig. S2. Example of OxCal modeled petrographic boundary. A: real color scanned image of core F (+3.1) between 62 and 312 mm depth from top. **B**: annotated drawing distinguishing folia calcite (deposited at the water table) from mammillary calcite (deposited below the water table). **C**: OxCal age model (*19*) plotted with uncertainties.

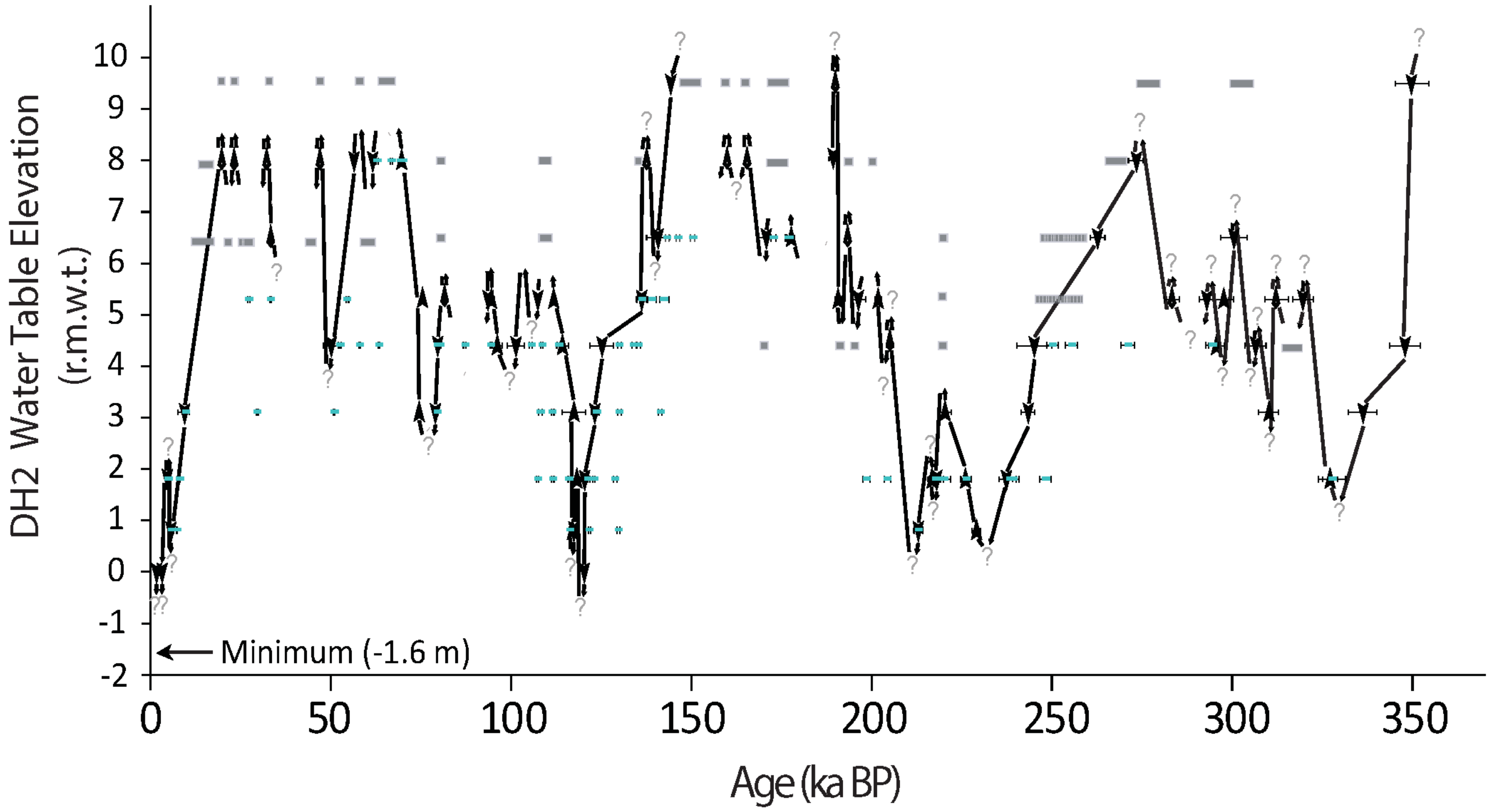

Fig. S3. DH2 water table record including nonextrapolated U-Th ages. 72 petrographic boundaries (black) with 2σ uncertainties. 25 estimated ages of thin mammillary calcite layers (grey). U-Th dates used to extrapolate the age of petrographic boundaries (blue) with 2σ uncertainties. Because all U-Th dates are drilled in mammillary (sub-aqueous) calcite, the presence of a blue point represents the timing of when the water table was above that specific elevation. Dashed horizontal bars represent estimated water-table minimums, as determined by the presence of thin mammillary calcite layers (see text). Question marks denote uncertainties in water-table minimums/maximums.

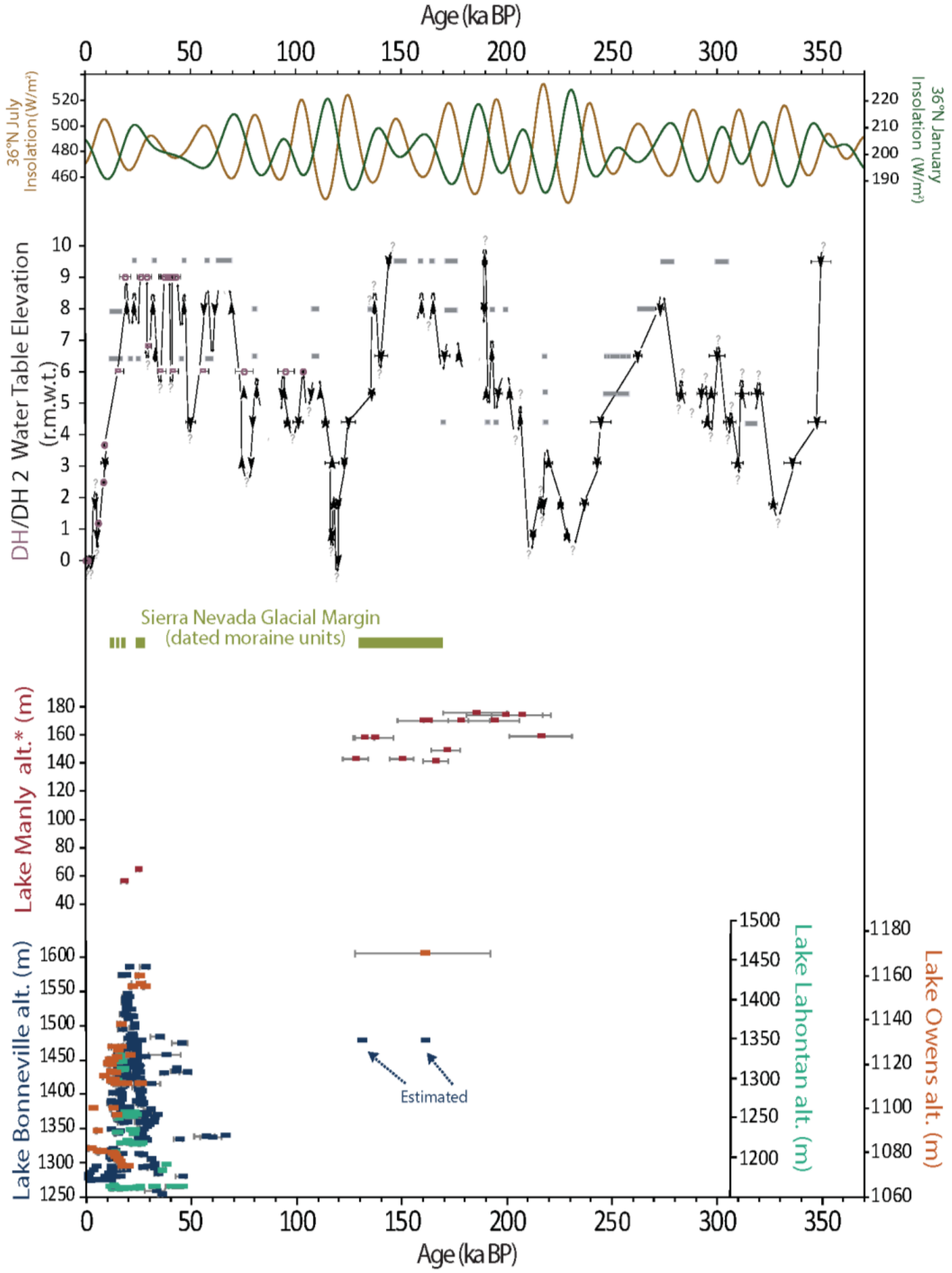

Fig. S4. Past regional moisture availability recorded in the GB. From the top: 36°N insolation in January (green) and July (yellow) (*24*). Water-table fluctuations recorded in Devils Hole (purple, *11*) and Devils Hole 2 (black, this study). Sierra Nevada glacial margins (*23*). Paleo-lake level altitudes from Lake Manly (red, *37*) adjusted relative to modern day. Paleo-lake level altitudes from Lake Lahontan (turquoise, *34*), Lake Owens (orange, *42*), and Lake Bonneville (blue, *43*, *44*). Lake level measurements are reported with 2σ uncertainty, unless estimated.

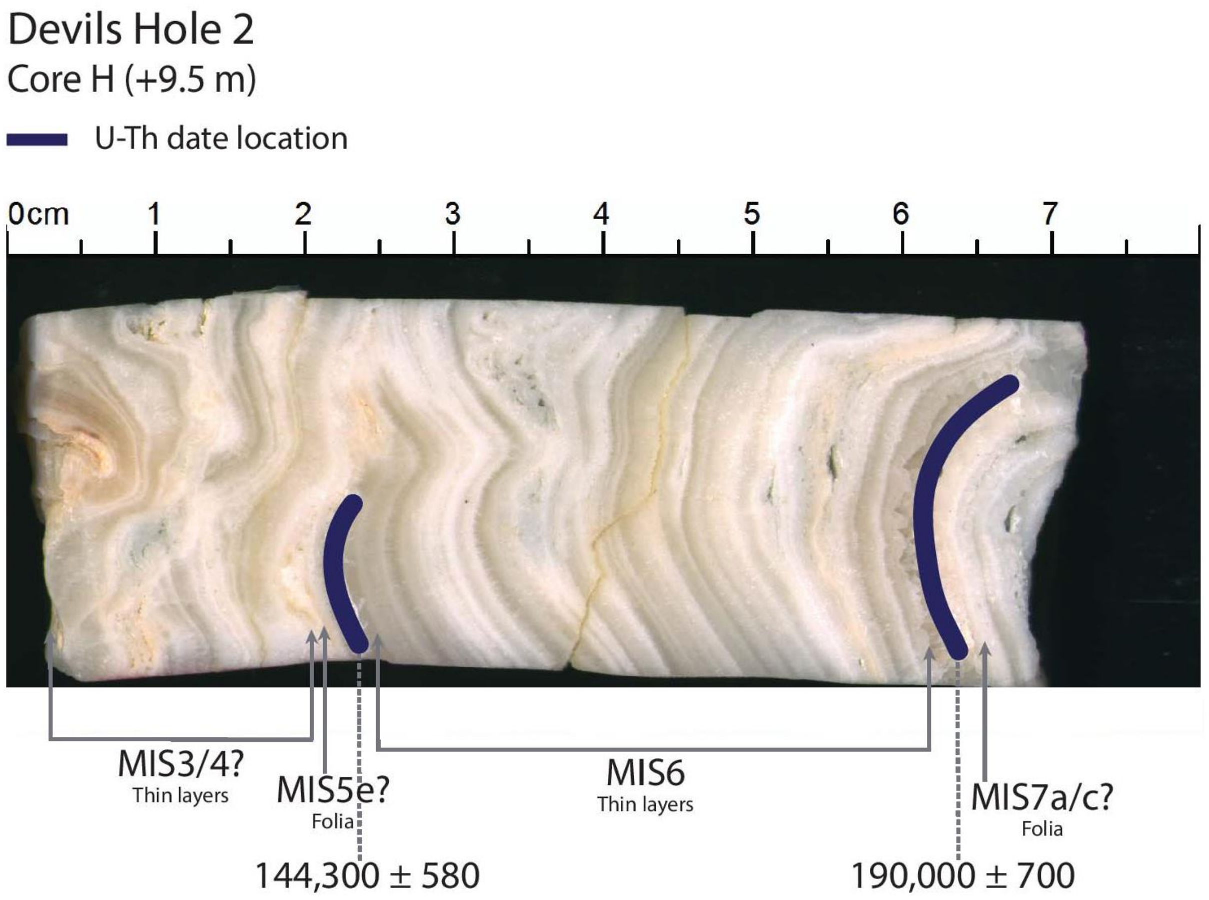

Fig. S5. Real-color scanned image of core H collected at +9.5 m r.m.w.t. Location of U-Th drill locations indicated in dark blue, with measured age reported to 2σ uncertainty. A total of four mammillary calcite layers were deposited between 144.3 ka and the termination of deposition. These four mammillary calcite layers (formed sub-aqueously) were likely deposited during MIS 4/3, when the water table is recorded to be higher than +8 m r.m.w.t. (see main text). Between 190.0 to 144.3 ka, over ten separate mammillary calcite layers were deposited. Based on this evidence, we conclude that the AMGFS water table maintained heights at or above +9.5m for increased periods of time during MIS 6, as opposed to MIS 4/3.

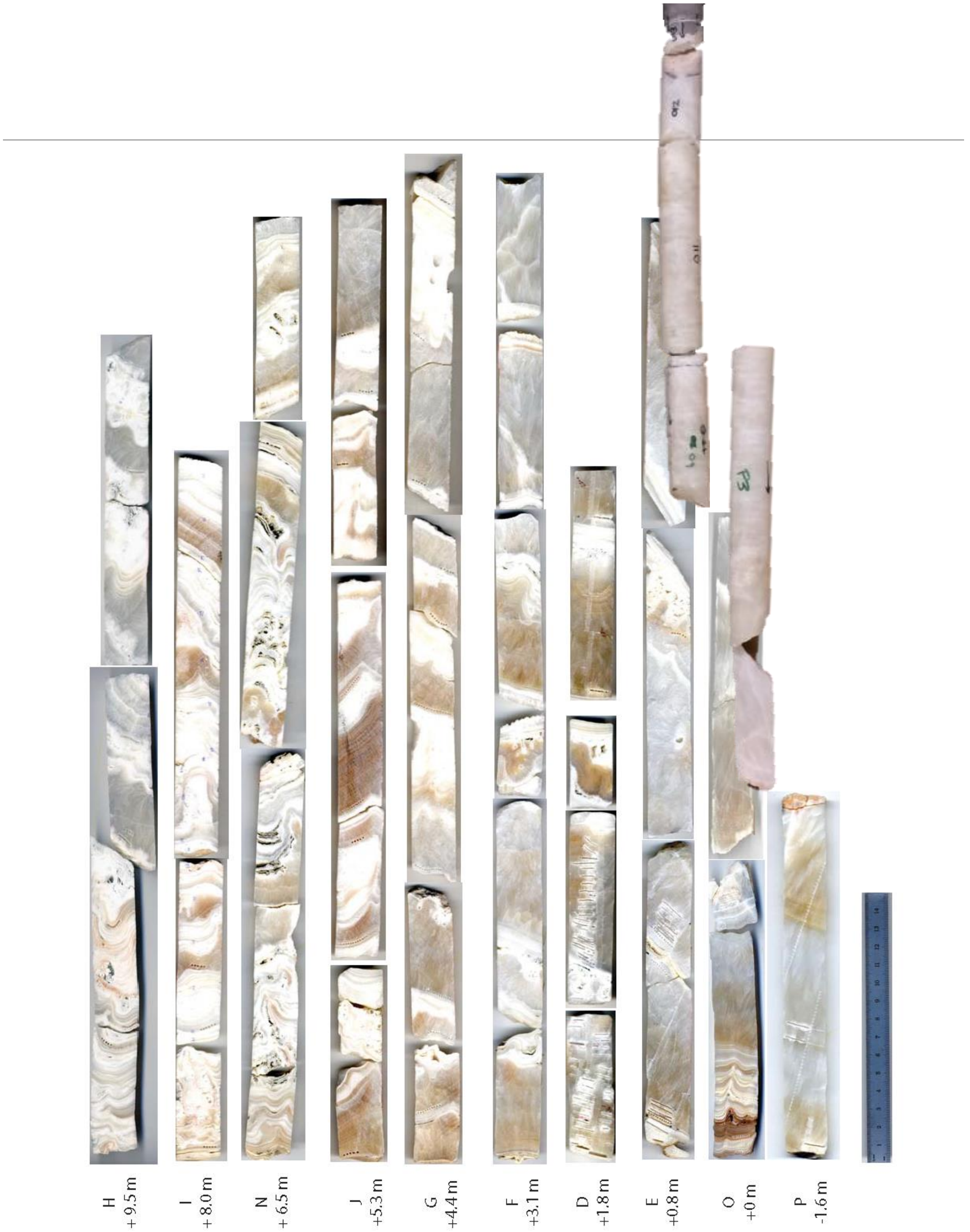

Fig. S6. Real-color scanned image of all DH2 cores included in this study. This includes the uncut cores from +0 and -1.6 m r.m.w.t. that are comprised entirely of mammillary calcite.

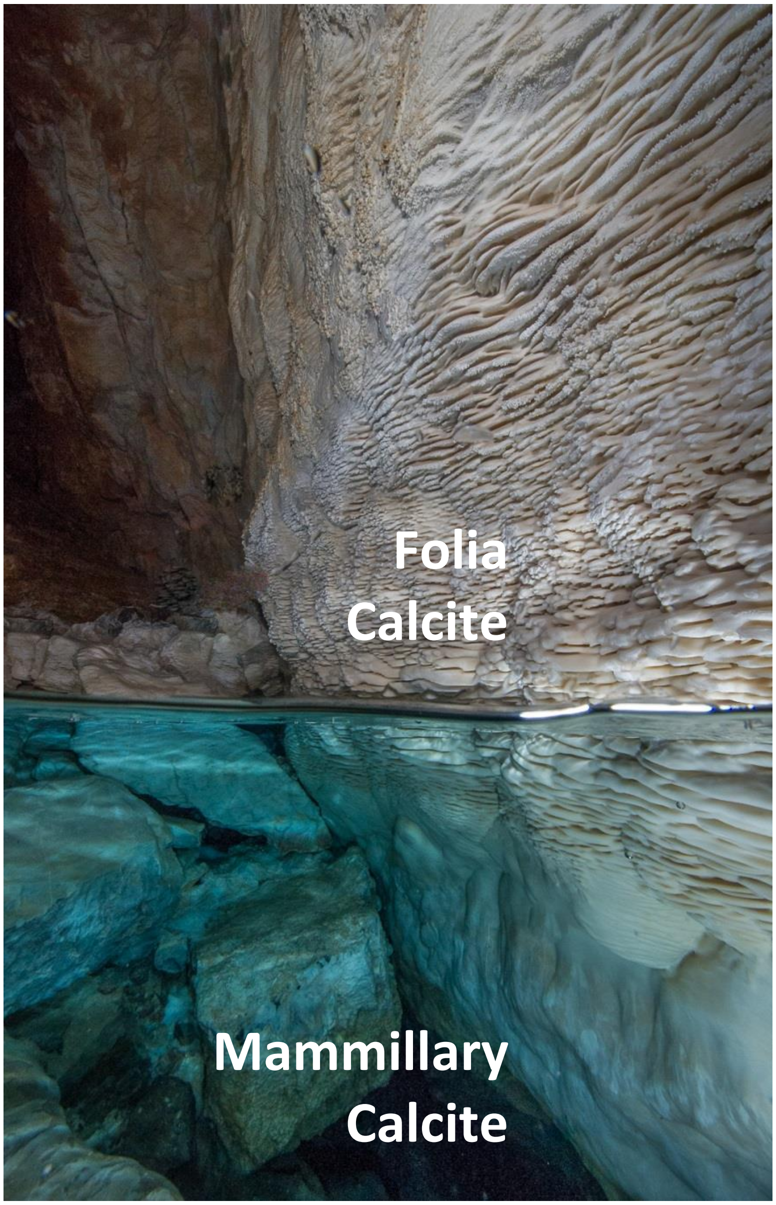

Fig. S7. Photo of folia and mammillary calcite in DH cave. Image courtesy of B. Seymour in association with the US National Parks Services. Width of image in foreground c. 0.7m.

Table S1. U-Th dating results. Analytical errors are 2σ of the mean. DFT = distance from top of core. U decay constants (*40*, *45*): λ_{238} = 1.55125x10^{-10} and λ_{234} = 2.82206x10^{-6}. Th decay constant (*40*): λ_{230} = 9.1705x10^{-6}. Age corrections were calculated using an average crustal $^{230}Th/^{232}Th$ atomic ratio of 4.4 x 10^{-6} ± 2.2 x 10^{-6}. Selected values represent material at secular equilibrium, with the crustal $^{232}Th/^{238}U$ value of 3.8. *$\delta^{234}U = ([^{234}U/^{238}U]_{activity} - 1) \times 1000$. ** $\delta^{234}U_{initial}$ values were back-calculated based on respective U-Th corrected age. ***B.P. stands for "Before Present" where the "Present" is defined as the year 1950 A.D.

	Elevation	dft	Description	^{238}U (ppb)		^{232}Th (ppt)		^{230}Th / ^{232}Th (atomic x10-6)		$\delta^{234}U$* (measured)		^{230}Th / ^{238}U (activity)		^{230}Th Age (yr) (uncorrected)		^{230}Th Age (yr) (corrected)		$\delta^{234}U$Initial** (corrected)		**^{230}Th Age (yr BP)*** (corrected)**	
**Core O**	0	0.5	Folia																		
	0	7.9	Calcite layers too thin to date																		
	0	8.5	Folia																		
	0	17.6	Calcite layers too thin to date																		
	0	21	Folia																		
	0	21.5	Mammillary Calcite	1516.4	±4.2	1394	±33	841	±25	1746.5	±4.1	0.0469	±0.0008	1876	±34	1866	±35	1756	±4	**1800**	**±35**
	0	30	Folia																		
	0	31	Mammillary Calcite	37602.3	±50.8	105450	±2115	419	±9	1745.2	±2.5	0.0712	±0.0003	2859	±13	2830	±25	1759	±3	**2765**	**±25**
	0	42	Folia																		
	0	44	Undatable calcite layer																		
	0	138	Folia																		
	0	139	Mammillary Calcite	18923.3	±3.3	24	±3	2035236	±289622	1163.5	±2.4	1.5668	±0.0031	120428	±451	120428	±451	1634	±4	**120350**	**±450**
	0	142	Mammillary Calcite	668.8	±1.0	543	±19	32539	±1117	1118.5	±2.2	1.6010	±0.0036	129555	±543	129546	±543	1612	±4	**129500**	**±540**
	0	145	Mammillary Calcite	471.1	±0.5	1137	±24	11598	±245	1171.8	±2.2	1.6981	±0.0028	136928	±473	136902	±473	1724	±4	**136840**	**±470**
**Core E**	0.8	0.3	Folia																		
	0.8	0.4	Mammillary Calcite	1526.4	±2.1	2557	±52	1392	±28	1713.5	±2.4	0.1414	±0.0004	5806	±16	5788	±21	1742	±2	**5720**	**±20**
	0.8	5.4	Mammillary Calcite	1512.9	±2.1	1241	±25	3058	±62	1728.4	±2.6	0.1522	±0.0003	6223	±15	6214	±16	1759	±3	**6150**	**±15**
	0.8	9.9	Mammillary Calcite	1521.2	±2.0	2025	±41	2177	±44	1715.7	±2.3	0.1757	±0.0004	7247	±17	7233	±20	1751	±2	**7170**	**±20**
	0.8	81.4	Mammillary Calcite	948.3	±1.0	293	±7	82839	±2036	1187.5	±2.0	1.5515	±0.0024	116313	±339	116310	±339	1649	±3	**116250**	**±340**
	0.8	89.90	Folia																		
	0.8	91.9	Mammillary Calcite	1694.0	±2.8	93	±5	463452	±22717	1152.9	±2.5	1.5361	±0.0037	117615	±508	117614	±508	1607	±4	**117550**	**±510**

	Elevation	dft	Description	^{238}U (ppb)		^{232}Th (ppt)		^{230}Th / ^{232}Th (atomic x10-6)		$\delta^{234}U$* (measured)		^{230}Th / ^{238}U (activity)		^{230}Th Age (yr) (uncorrected)		^{230}Th Age (yr) (corrected)		$\delta^{234}U$Initial** (corrected)		**^{230}Th Age (yr BP)*** (corrected)**	
	0.8	96.9	Mammillary Calcite	549.0	±0.5	105	±3	137978	±4298	1191.4	±1.8	1.5987	±0.0024	121709	±349	121707	±349	1680	±3	**121640**	**±350**
	0.8	98.9	Mammillary Calcite	562.0	±0.6	1021	±21	14573	±296	1122.8	±2.1	1.6061	±0.0028	129762	±449	129742	±449	1619	±4	**129680**	**±450**
	0.8	222.4	Folia																		
	0.8	222.9	Mammillary Calcite	1619.3	±2.6	22	±3	2275966	±318718	961.5	±2.1	1.8877	±0.0036	212974	±1183	212973	±1183	1754	±7	**212900**	**±1200**
	0.8	248.2.4	Mammillary Calcite	471.7	±0.5	7316	±147	2029	±41	930.2	±2.2	1.9082	±0.0029	229297	±1215	229133	±1219	1776	±7	**229100**	**±1200**
	0.8	285.4	Folia																		
**Core D**	1.8	0	Mammillary Calcite	1906.5	±2.0	10850	±218	356	±7	1716.9	±2.4	0.1228	±0.0003	5021	±15	4961	±45	1741	±2	**4895**	**±45**
	1.8	0.9	Mammillary Calcite	1718.3	±3.6	4574	±100	1203	±28	1713.2	±2.4	0.1942	±0.0017	8038	±72	8010	±75	1752	±2	**7945**	**±75**
	1.8	2.3	Mammillary Calcite	1502.4	±1.6	16147	±324	303	±6	1707.5	±2.8	0.1977	±0.0005	8205	±23	8091	±84	1747	±3	**8025**	**±85**
	1.8	65.4	Mammillary Calcite	425.8	±0.4	9407	±188	1139	±23	1257.9	±2.1	1.5265	±0.0028	107590	±338	107352	±377	1703	±3	**107290**	**±380**
	1.8	69.4	Mammillary Calcite	415.8	±0.4	244	±7	43463	±1223	1236.7	±2.0	1.5470	±0.0028	111552	±356	111545	±356	1694	±3	**111480**	**±360**
	1.8	77.7	Mammillary Calcite	602.4	±0.7	123	±7	126477	±7194	1204.8	±2.2	1.5630	±0.0031	116139	±415	116137	±415	1672	±4	**116070**	**±420**
	1.8	95.1	Folia																		
	1.8	97.4	Mammillary Calcite	1054.4	±1.3	91	±3	297873	±8494	1153.2	±2.3	1.5671	±0.0027	121465	±411	121464	±411	1625	±4	**121410**	**±410**
	1.8	99.4	Mammillary Calcite	511.3	±0.4	72	±3	185162	±6478	1163.2	±1.7	1.5864	±0.0020	122928	±304	122926	±304	1646	±3	**122860**	**±310**
	1.8	101.4	Mammillary Calcite	564.0	±0.6	533	±11	27891	±595	1123.2	±2.1	1.5995	±0.0026	128826	±421	128816	±421	1616	±4	**128750**	**±420**
	1.8	155.4	Mammillary Calcite	407.6	±0.4	1837	±37	6946	±140	1024.8	±2.2	1.8987	±0.0031	198544	±936	198497	±937	1794	±6	**198440**	**±940**
	1.8	158.4	Mammillary Calcite	430.3	±0.3	1879	±38	7208	±146	1012.1	±1.9	1.9092	±0.0030	204412	±918	204366	±918	1802	±6	**204300**	**±920**
	1.8	169.8	Mammillary Calcite	852.5	±1.6	3347	±68	8042	±164	970.5	±2.6	1.9151	±0.0062	217974	±1966	217932	±1966	1795	±11	**217900**	**±1200**
	1.8	187.4	Folia																		
	1.8	192.6	Mammillary Calcite	424.7	±0.5	1979	±40	6722	±137	957.2	±1.9	1.9002	±0.0040	217856	±1307	217806	±1307	1770	±7	**217740**	**±1310**
	1.8	203.6	Mammillary Calcite	521.1	±0.7	7272	±146	2232	±45	939.2	±2.1	1.8889	±0.0051	220263	±1678	220115	±1680	1748	±9	**220050**	**±1680**

	Elevation	dft	Description	^{238}U (ppb)		^{232}Th (ppt)		^{230}Th / ^{232}Th (atomic x10-6)		$\delta^{234}U$* (measured)		^{230}Th / ^{238}U (activity)		^{230}Th Age (yr) (uncorrected)		^{230}Th Age (yr) (corrected)		$\delta^{234}U$Initial** (corrected)		**^{230}Th Age (yr BP)*** (corrected)**	
	1.8	208.6	Mammillary Calcite	407.6	±0.5	3180	±64	3992	±81	921.7	±2.2	1.8891	±0.0044	226235	±1571	226151	±1571	1745	±9	**226090**	**±1570**
	1.8	229	Folia																		
	1.8	230	Mammillary Calcite	506.4	±0.6	2367	±48	6666	±136	889.0	±2.0	1.8893	±0.0043	238447	±1711	238397	±1711	1742	±9	**238330**	**±1710**
	1.8	231.6	Mammillary Calcite	498.7	±0.6	4419	±89	3498	±70	878.3	±2.0	1.8797	±0.0036	239347	±1541	239252	±1541	1725	±8	**239190**	**±1540**
	1.8	233.4	Mammillary Calcite	551.0	±0.6	9039	±181	1907	±38	872.3	±2.1	1.8972	±0.0029	248412	±1476	248238	±1479	1757	±9	**248180**	**±1480**
	1.8	300	Mammillary Calcite	153.9	±0.1	276	±8	16777	±512	692.4	±1.8	1.8259	±0.0041	327802	±3779	327781	±3778	1746	±19	**327720**	**±3780**
	1.8																				
**Core F**	3.1	3	Folia																		
	3.1	3.2	Mammillary Calcite	1419.0	±3.3	1187	±25	4571	±97	1686.7	±3.1	0.2320	±0.0009	9760	±43	9751	±43	1734	±3	**9685**	**±40**
	3.1	7	Mammillary Calcite	449.4	±0.3	10196	±204	476	±10	1674.4	±2.3	0.6549	±0.0018	29744	±97	29509	±192	1820	±3	**29440**	**±190**
	3.1	12	Mammillary Calcite	450.0	±0.3	5073	±102	1467	±30	1576.9	±2.2	1.0030	±0.0025	50965	±162	50849	±182	1820	±3	**50780**	**±180**
	3.1	49	Mammillary Calcite	366.4	±0.4	542	±11	17312	±353	1976.4	±2.9	1.5521	±0.0023	73326	±172	73313	±173	2431	±4	**73250**	**±170**
	3.1	73	Folia																		
	3.1	75	Mammillary Calcite	411.5	±0.4	197	±7	53155	±1975	1781.6	±2.6	1.5399	±0.0025	79658	±202	79654	±202	2231	±3	**79590**	**±200**
	3.1	79	Mammillary Calcite	381.4	±0.3	678	±15	14183	±306	1255.9	±2.3	1.5297	±0.0035	108098	±418	108079	±418	1704	±4	**108010**	**±420**
	3.1	85	Mammillary Calcite	411.7	±0.3	415	±10	25210	±624	1232.5	±2.2	1.5428	±0.0027	111425	±354	111414	±354	1688	±3	**111350**	**±350**
	3.1	90.9	Mammillary Calcite																		
	3.1	111	Folia																		
	3.1	112	Mammillary Calcite	1767.7	±3.8	28	±9	1660512	±549231	1143.8	±2.4	1.5776	±0.0052	123741	±714	123740	±714	1622	±5	**123670**	**±710**
	3.1	118.5	Mammillary Calcite	562.9	±0.6	1444	±29	10365	±209	1127.3	±2.0	1.6124	±0.0029	130129	±454	130100	±454	1627	±4	**130030**	**±450**
	3.1	121.5	Mammillary Calcite	542.4	±0.6	1092	±22	14125	±285	1166.5	±2.1	1.7245	±0.0026	141340	±455	141319	±455	1738	±4	**141250**	**±460**
	3.1	186.5	Folia																		

	Elevation	dft	Description	^{238}U (ppb)		^{232}Th (ppt)		^{230}Th / ^{232}Th (atomic x10-6)		$\delta^{234}U$* (measured)		^{230}Th / ^{238}U (activity)		^{230}Th Age (yr) (uncorrected)		^{230}Th Age (yr) (corrected)		$\delta^{234}U$Initial** (corrected)		**^{230}Th Age (yr BP)*** (corrected)**	
	3.1	213	Mammillary Calcite	444.9	±0.6	948	±21	14742	±326	953.5	±2.1	1.9057	±0.0048	220612	±1570	220589	±1570	1777	±9	**220500**	**±1600**
	3.1	248	Folia																		
	3.1	248.5	Mammillary Calcite	491.9	±0.6	3402	±68	4517	±91	881.5	±2.3	1.8950	±0.0043	243578	±1864	243505	±1864	1752	±10	**243400**	**±1900**
	3.1	279	Mammillary Calcite	243.1	±0.2	4069	±82	1839	±37	755.7	±1.9	1.8668	±0.0026	300973	±2160	300795	±2161	1766	±12	**300700**	**±2200**
	3.1	320	Folia																		
	3.1	323	Mammillary Calcite	43.7	±0.1	55	±1	23444	±535	661.4	±2.0	1.7966	±0.0038	336239	±3987	336224	±3987	1708	±20	**336200**	**±4000**
**Core G**	4.4	0.5	Folia																		
	4.4	2.5	Mammillary Calcite	438.1	±0.5	13329	±267	552	±11	1541.1	±2.3	1.0178	±0.0017	52801	±126	52483	±258	1787	±3	**52420**	**±260**
	4.4	5.5	Mammillary Calcite	447.9	±0.5	8313	±167	978	±20	1541.1	±2.3	1.1003	±0.0019	58139	±141	57947	±196	1815	±3	**57880**	**±200**
	4.4	10	Mammillary Calcite	459.1	±0.5	5386	±108	1634	±33	1506.3	±2.4	1.1628	±0.0021	63465	±170	63343	±190	1801	±3	**63280**	**±190**
	4.4	18.5	Mammillary Calcite	234.1	±0.2	6687	±134	785	±16	1462.0	±2.2	1.3589	±0.0020	79840	±192	79546	±282	1830	±3	**79480**	**±280**
	4.4	26	Folia																		
	4.4	31	Mammillary Calcite	427.5	±0.4	12254	±246	791	±16	1476.8	±2.2	1.3755	±0.0023	80490	±212	80197	±296	1852	±3	**80130**	**±300**
	4.4	34	Mammillary Calcite	412.3	±0.4	6722	±135	1416	±28	1380.9	±2.2	1.4001	±0.0028	87338	±274	87167	±299	1766	±3	**87100**	**±300**
	4.4	43	Mammillary Calcite	352.9	±0.3	695	±14	12117	±248	1332.6	±2.4	1.4482	±0.0022	94512	±256	94491	±257	1740	±3	**94430**	**±260**
	4.4	64	Folia																		
	4.4	68	Mammillary Calcite	376.0	±0.4	180	±5	52378	±1418	1274.2	±2.7	1.5188	±0.0027	105533	±345	105528	±345	1716	±4	**105460**	**±350**
	4.4	71	Mammillary Calcite	346.5	±0.4	453	±10	19352	±432	1256.0	±2.3	1.5342	±0.0029	108583	±363	108569	±363	1706	±4	**108500**	**±360**
	4.4	75	Mammillary Calcite	313.3	±0.3	311	±8	25842	±627	1230.1	±2.2	1.5565	±0.0033	113186	±416	113175	±417	1693	±4	**113110**	**±420**
	4.4	87	Folia																		
	4.4	87.8	Mammillary Calcite	1394.0	±1.7	63	±4	583060	±40111	1122.2	±2.0	1.6075	±0.0027	130023	±440	130023	±440	1620	±4	**129960**	**±440**

	Elevation	dft	Description	^{238}U (ppb)		^{232}Th (ppt)		^{230}Th / ^{232}Th (atomic x10-6)		$\delta^{234}U$* (measured)		^{230}Th / ^{238}U (activity)		^{230}Th *Age (yr)* (uncorrected)		^{230}Th *Age (yr)* (corrected)		$\delta^{234}U$*Initial*** (corrected)		**^{230}Th *Age (yr BP)**** (corrected)**	
	4.4	89	Mammillary Calcite	571.4	±0.7	4407	±89	3505	±71	1125.4	±2.3	1.6395	±0.0032	134118	±528	134033	±531	1643	±4	**133970**	**±530**
	4.4	92	Mammillary Calcite	536.6	±0.6	5416	±109	2740	±55	1158.8	±2.1	1.6769	±0.0031	135483	±492	135374	±498	1698	±4	**135310**	**±500**
	4.4	145	Folia																		
	4.4	158																			
	4.4	182	Folia																		
	4.4	183	Thin Mammillary Calcite	469.5	±0.5	507	±10	28730	±580	979.1	±2.0	1.8830	±0.0029	206631	±954	206619	±953	1754	±6	**206550**	**±950**
	4.4	235																			
	4.4	237	Mammillary Calcite	312.5	±0.3	6770	±136	1450	±29	874.5	±2.2	1.9047	±0.0033	250331	±1621	250103	±1626	1771	±9	**250000**	**±1600**
	4.4	240	Mammillary Calcite	423.5	±0.5	3048	±61	4360	±88	861.7	±2.0	1.9029	±0.0033	255534	±1675	255457	±1675	1772	±9	**255400**	**±1700**
	4.4	246	Mammillary Calcite	507.7	±0.5	5915	±119	2708	±54	839.9	±2.0	1.9135	±0.0032	271284	±1880	271162	±1880	1805	±11	**271100**	**±1900**
	4.4	256	Mammillary Calcite	349.4	±0.3	1028	±21	11723	±237	949.1	±2.0	2.0925	±0.0025	294532	±1759	294504	±1759	2179	±12	**294400**	**±1800**
	4.4	283	Folia																		
	4.4	285.5	Mammillary Calcite	466.1	±0.5	2397	±48	6160	±124	792.7	±2.3	1.9215	±0.0036	306655	±2871	306602	±2870	1883	±16	**306500**	**±2900**
	4.4	314	Folia																		
	4.4	330.7	Undatable calcite layer																		
	4.4	342	Folia																		
	4.4	343	Mammillary Calcite	873.7	±1.1	90	±3	291464	±10777	666.4	±2.1	1.8158	±0.0034	348032	±4185	348030	±4185	1779	±22	**348000**	**±4200**
**Core J**	5.3	2	Mammillary Calcite	480.4	±0.4	18118	±363	269	±5	1685.1	±2.4	0.6151	±0.0009	27612	±53	27222	±281	1820	±3	**27160**	**±280**
	5.3	5	Mammillary Calcite	496.4	±0.5	13554	±272	439	±9	1662.2	±2.3	0.7272	±0.0016	33644	±89	33362	±218	1826	±3	**33300**	**±220**
	5.3	9.5	Mammillary Calcite	487.7	±0.5	17972	±360	473	±10	1558.9	±2.4	1.0579	±0.0018	54879	±132	54498	±300	1818	±3	**54430**	**±300**
	5.3	33	Mammillary Calcite	461.9	±0.4	33401	±669	301	±6	1478.1	±2.2	1.3204	±0.0020	76097	±183	75351	±558	1828	±4	**75290**	**±558**
	5.3	38	Folia																		

Elevation	dft	Description	^{238}U (ppb)		^{232}Th (ppt)		^{230}Th / ^{232}Th (atomic x10-6)		$\delta^{234}U$* (measured)		^{230}Th / ^{238}U (activity)		^{230}Th Age (yr) (uncorrected)		^{230}Th Age (yr) (corrected)		$\delta^{234}U$Initial** (corrected)		**^{230}Th Age (yr BP)*** (corrected)**	
5.3	38.2-39.1	Thin Mammillary Calcite	385.4	±0.4	18731	±375	463	±9	1423.3	±2.4	1.3650	±0.0032	82174	±288	81667	±459	1792	±4	**81600**	**±460**
5.3	102	Folia																		
5.3	102.1	Mammillary Calcite	1669.3	±2.9	340	±9	118052	±3042	1363.2	±2.4	1.4595	±0.0030	93708	±314	93706	±314	1776	±4	**93640**	**±320**
5.3	102.2-109																		**94760**	**±530**
5.3	111.5	Folia																		
5.3	143.8	Calcite layers too thin to date																		
5.3	158	Folia																		
5.3	158.1	Mammillary Calcite	408.6	±0.4	25674	±514	401	±8	1252.9	±2.6	1.5285	±0.0030	108195	±382	107515	±613	1697	±5	**107450**	**±610**
5.3	171.6	Mammillary Calcite	1475.4	±2.7	3134	±63	11924	±240	1217.4	±2.6	1.5362	±0.0032	111929	±419	111906	±419	1669	±4	**111840**	**±420**
5.3	188.7	Folia																		
5.3	189	Mammillary Calcite	650.3	±1.6	83690	±1683	217	±4	1157.0	±3.8	1.6913	±0.0066	137726	±1047	136332	±1429	1700	±9	**136300**	**±1400**
5.3	190	Mammillary Calcite	521.4	±1.2	9560	±192	1538	±31	1166.4	±3.9	1.7101	±0.0072	139261	±1130	139065	±1136	1727	±8	**139000**	**±1100**
5.3	192	Mammillary Calcite	505.5	±1.4	10133	±204	1421	±29	1159.7	±4.0	1.7272	±0.0077	142591	±1239	142377	±1246	1733	±9	**142300**	**±1300**
5.3	225	Mammillary Calcite	409.6	±0.4	11188	±224	1152	±23	1066.2	±2.1	1.9090	±0.0027	191088	±763	190807	±787	1827	±5	**190700**	**±790**
5.3	246	Folia																		
5.3	247-248.9	Mammillary Calcite	401.7	±0.4	14006	±281	907	±18	1051.1	±2.2	1.9182	±0.0032	196731	±917	196372	±948	1829	±6	**196310**	**±950**
5.3	267	Mammillary Calcite																	**201820**	**±900**
5.3	293	Folia																		
5.3	298	Calcite layers too thin to date																		
5.3	314.2	Folia																		
5.3	318	Thin Mammillary Calcite	2195.7	±2.0	102425	±2051	680	±14	826.4	±2.0	1.9227	±0.0033	283851	±2134	283371	±2153	1839	±12	**283300**	**±2200**

	Elevation	dft	Description	^{238}U (ppb)		^{232}Th (ppt)		^{230}Th / ^{232}Th (atomic x10-6)		$\delta^{234}U$* (measured)		^{230}Th / ^{238}U (activity)		^{230}Th Age (yr) (uncorrected)		^{230}Th Age (yr) (corrected)		$\delta^{234}U$Initial** (corrected)		**^{230}Th Age (yr BP)*** (corrected)**	
	5.3	332.5	Folia																		
	5.3	339	Calcite layers too thin to date																		
	5.3	344																			
	5.3	345	Mammillary Calcite	413.9	±0.4	6616	±132	1952	±39	787.4	±1.8	1.8925	±0.0027	293107	±2009	292940	±2010	1800	±11	**292900**	**±2000**
	5.3	351	Mammillary Calcite	321.6	±0.3	3685	±74	2701	±55	768.3	±1.8	1.8773	±0.0041	297875	±2774	297754	±2773	1780	±15	**297700**	**±2800**
	5.3	357	Folia																		
	5.3	358	Thin Mammillary Calcite	363.6	±0.4	7776	±156	1437	±29	739.1	±1.9	1.8640	±0.0040	312621	±3222	312396	±3220	1785	±17	**312300**	**±3200**
	5.3	374	Folia																		
	5.3	375	Mammillary Calcite	394.8	±0.4	1184	±24	10204	±206	724.5	±1.9	1.8558	±0.0031	319712	±2851	319680	±2850	1786	±15	**319600**	**±2900**
**Core N**	6.5	5	Calcite layers too thin to date																		
	6.5	10.7	Folia																		
	6.5	15.5	Undatable calcite layer																		
	6.5	30	Folia																		
	6.5	30.1-30.8	Thin Mammillary Calcite	1012.5	±1.4	4935	±100	2376	±48	1581.4	±2.7	0.7022	±0.0016	33519	±97	33467	±104	1738	±3	**33400**	**±110**
	6.5	30	Folia																		
	6.5	40.8	Calcite layers too thin to date																		
	6.5	118	Folia																		
	6.5	120.5	Mammillary Calcite	487.5	±0.6	435	±10	32015	±763	1163.0	±2.0	1.7344	±0.0029	143236	±507	143226	±507	1742	±4	**143160**	**±510**
	6.5	122.5	Mammillary Calcite	464.5	±0.5	330	±8	40268	±947	1141.2	±2.1	1.7354	±0.0029	146229	±524	146221	±524	1724	±4	**146160**	**±520**
	6.5	125	Mammillary Calcite	458.8	±0.5	330	±8	40259	±951	1137.2	±2.2	1.7584	±0.0033	150347	±610	150339	±610	1738	±5	**150270**	**±610**
	6.5	139.5	Folia																		

	Elevation	dft	Description	^{238}U (ppb)		^{232}Th (ppt)		^{230}Th / ^{232}Th (atomic x10-6)		$\delta^{234}U$* (measured)		^{230}Th / ^{238}U (activity)		^{230}Th *Age (yr)* (uncorrected)		^{230}Th *Age (yr)* (corrected)		$\delta^{234}U$*Initial*** (corrected)		**^{230}Th *Age (yr BP)**** (corrected)**	
	6.5	140.5	Mammillary Calcite	532.2	±0.6	582	±12	27729	±592	1084.5	±2.1	1.8387	±0.0029	172822	±680	172810	±680	1766	±5	**172750**	**±680**
	6.5	145	Mammillary Calcite	510.0	±0.6	1098	±22	14292	±291	1089.3	±2.1	1.8659	±0.0027	177227	±675	177205	±675	1796	±5	**177140**	**±660**
	6.5	152.5	Mammillary Calcite	424.3	±0.4	1657	±33	8758	±177	1305.0	±2.1	2.0746	±0.0038	176927	±755	176891	±755	2150	±6	**176830**	**±760**
	6.5	165.5	Folia																		
	6.5	165.7-166.4	Thin Mammillary Calcite	327.4	±0.7	119	±7	86167	±5406	1049.5	±4.9	1.9018	±0.0066	193316	±1901	193312	±1901	1811	±13	**193300**	**±1900**
	6.5	183.5	Folia																		
	6.5	185-202.5	Undatable calcite layer																		
	6.5	244	Folia																		
	6.5	253	Undatable calcite layer																		
	6.5	362	Folia																		
	6.5	365	Undatable calcite layer																		
	6.5	367	Folia																		
	6.5	379	Calcite layers too thin to date																		
	6.5	420	Folia																		
	6.5	422	Mammillary Calcite	806.5	±1.0	10214	±205	2469	±50	841.3	±1.8	1.8963	±0.0040	262923	±2011	262789	±2012	1766	±11	**262700**	**±2000**
	6.5	473	Folia																		
	6.5	475	Mammillary Calcite	570.2	±0.8	4965	±100	3571	±72	771.7	±2.2	1.8860	±0.0054	300651	±3720	300560	±3718	1802	±20	**300500**	**±3700**
	6.5	503.5	Folia																		
**Core I**	8	5	Calcite layers too thin to date																		
	8	6	Folia																		
	8	6.3	Thin Mammillary Calcite	1230.3	±1.4	1869	±38	4948	±100	1681.6	±2.4	0.4558	±0.0008	19933	±40	19917	±42	1779	±3	**19850**	**±40**

Elevation	dft	Description	^{238}U (ppb)		^{232}Th (ppt)		^{230}Th / ^{232}Th (atomic x10-6)		$\delta^{234}U$* (measured)		^{230}Th / ^{238}U (activity)		^{230}Th Age (yr) (uncorrected)		^{230}Th Age (yr) (corrected)		$\delta^{234}U$Initial** (corrected)		**^{230}Th Age (yr BP)*** (corrected)**	
8	8	Folia																		
8	24	Calcite layers too thin to date																		
8	32	Folia																		
8	34	Thin Mammillary Calcite	1419.8	±2.3	581	±12	37962	±807	1586.6	±2.7	0.9428	±0.0019	47086	±126	47082	±126	1812	±3	**47020**	**±130**
8	45	Folia																		
8	59	Calcite layers too thin to date																		
8	70.6	Folia																		
8	70.7	Mammillary Calcite	1411.0	±1.8	350	±7	71107	±1489	1522.4	±2.5	1.0690	±0.0019	56614	±145	56611	±145	1786	±3	**56550**	**±150**
8	77	Folia																		
8	103.3	Calcite layers too thin to date																		
8	104	Folia																		
8	105	Mammillary Calcite	539.2	±0.5	7212	±145	1423	±29	1505.7	±2.6	1.1545	±0.0018	62905	±153	62766	±182	1798	±3	**62700**	**±180**
8	107.5	Mammillary Calcite	453.8	±0.4	11120	±223	815	±16	1501.8	±2.6	1.2112	±0.0017	67040	±154	66786	±236	1813	±3	**66720**	**±240**
8	108.5	Mammillary Calcite	395.2	±0.4	6149	±123	1403	±28	1635.2	±2.4	1.3235	±0.0023	70160	±181	70008	±210	1992	±3	**69940**	**±210**
8	110.5																			
8	133	Calcite layers too thin to date																		
8	134																			
8	135	Mammillary Calcite	475.4	±6.0	13756	±176	708	±2	1494.4	±2.1	1.2428	±0.0015	69602	±137	69303	±252	1817	±3	**69240**	**±250**
8	145	Folia																		
8	165	Calcite layers too thin to date																		

	Elevation	dft	Description	*^{238}U* (ppb)		*^{232}Th* (ppt)		*^{230}Th / ^{232}Th* (atomic x10-6)		*$\delta^{234}U$** (measured)		*^{230}Th / ^{238}U* (activity)		*^{230}Th Age (yr)* (uncorrected)		*^{230}Th Age (yr)* (corrected)		*$\delta^{234}U$Initial*** (corrected)		***^{230}Th Age (yr BP)****** **(corrected)**	
	8	181	Folia																		
	8	165	Calcite layers too thin to date																		
	8	212	Folia																		
	8	259	Calcite layers too thin to date																		
	8	265	Folia																		
	8	266	Thin Mammillary Calcite	955.1	±1.3	1042	±22	25792	±534	1175.2	±2.1	1.7064	±0.0031	137687	±502	137675	±502	1733	±4	**137610**	**±500**
	8	305	Folia																		
	8	306	Thin Mammillary Calcite	552.4	±0.6	200	±6	83026	±2614	1106.1	±2.2	1.8194	±0.0032	165417	±685	165413	±685	1764	±5	**165350**	**±690**
	8	315	Folia																		
	8	336	Calcite layers too thin to date																		
	8	337	Folia																		
	8	338	Mammillary Calcite	615.3	±0.8	14231	±286	1367	±28	1081.7	±2.1	1.9172	±0.0052	189442	±1214	189205	±1223	1845	±7	**189100**	**±1200**
	8	27	Calcite layers too thin to date																		
	8	28	Folia																		
**Core I- second half**	8	28.2-34.8	Thin Mammillary Calcite	1367.6	±2.0	886	±18	13387	±271	1680.2	±2.6	0.5259	±0.0009	23288	±51	23282	±52	1794	±3	**23215**	**±50**
	8	37	Folia																		
	8	37.2 - 38.9	Thin Mammillary Calcite	1566.8	±2.5	5282	±106	3429	±69	1657.7	±2.6	0.7012	±0.0014	32345	±82	32310	±85	1816	±3	**32245**	**±85**
	8	40	Folia																		
	8	428	Calcite layers too thin to date																		
	8	430	Folia																		

	Elevation	dft	Description	^{238}U (ppb)		^{232}Th (ppt)		^{230}Th / ^{232}Th (atomic x10-6)		$\delta^{234}U$* (measured)		^{230}Th / ^{238}U (activity)		^{230}Th *Age (yr)* (uncorrected)		^{230}Th *Age (yr)* (corrected)		$\delta^{234}U$*Initial*** (corrected)		**^{230}Th *Age (yr BP)**** (corrected)**	
	8	430.5	Thin Mammillary Calcite	521.9	±0.5	2588	±52	6036	±122	1135.0	±2.0	1.8153	±0.0036	159996	±690	159944	±690	1783	±5	**159880**	**±690**
	8	431	Folia																		
	8	633	Calcite layers too thin to date																		
	8	634	Folia																		
	8	635	Mammillary Calcite	497.7	±0.5	20158	±404	782	±16	841.7	±2.0	1.9207	±0.0034	273723	±1984	273304	±2000	1820	±11	**273200**	**±2000**
	8		Folia																		
**Core H**	9.5	15	Calcite layers too thin to date																		
	9.5	18	Folia																		
	9.5	19	Mammillary Calcite	818.2	±1.3	931	±20	27128	±573	1312.0	±2.5	1.8715	±0.0036	144402	±576	144390	±576	1972	±5	**144320**	**±580**
	9.5	20	Folia																		
	9.5	55	Calcite layers too thin to date																		
	9.5	59	Folia																		
	9.5	60	Thin Mammillary Calcite	429.9	±5.7	2502	±34	5466	±13	1091.1	±2.2	1.9296	±0.0023	190082	±694	190024	±695	1865	±5	**189960**	**±700**
	9.5	77	Calcite layers too thin to date																		
	9.5	78	Folia																		
	9.5	129	Calcite layers too thin to date																		
	9.5	129.5	Folia																		
	9.5	131	Mammillary Calcite	1068.6	±15.2	9091	±130	4028	±10	2331.6	±31.8	2.0781	±0.0044	350082	±4685	350012	±4682	2332	±32	**345000**	**±4700**
	9.5	176	Folia																		

Table S2. Age and location of petrographic boundaries. Extrapolated boundary ages in bold.

Elevation	dft	Increasing water table		Decreasing water table		Brief rises	
0	21			1800	±35		
0	30			2765	±25		
0	138			120360	±450		
0.8	0.3			**5690**	**±80**		
0.8	81.4	**116890**	**±400**				
0.8	89.90			117550	±510		
0.8	222.4			212910	±1180		
0.8	248.2.4	229100	±1200				
1.8	0			4896	±45		
1.8	77.7	**118300**	**±1300**				
1.8	95.1			**120300**	**±1000**		
1.8	169.8	**216750**	**±740**				
1.8	187.4			**217390**	**±500**		
1.8	208.6	**226050**	**±1160**				
1.8	229			**237200**	**±1900**		
1.8	300	**327200**	**±2000**				
3.1	3			**9200**	**±1500**		
3.1	49	**74410**	**±400**				
3.1	73			**78910**	**±370**		
3.1	90.9	**117500**	**±3300**				
3.1	111			**123280**	**±970**		
3.1	213	**220523**	**±1570**				
3.1	248			**243400**	**±1900**		
3.1	279	**310100**	**±2800**				
3.1	320			336200	±4000		
4.4	0.5			**50180**	**±2300**		
4.4	18.5	**79580**	**±180**				
4.4	26			**79770**	**±200**		
4.4	43	**96049**	**±1451**				
4.4	64			**101300**	**±2400**		
4.4	75	**114300**	**±1600**				
4.4	87			**125100**	**±3300**		
4.4	183					206550	±950
4.4	235			**245100**	**±4800**		
4.4	256	**295668**	**±2262**				
4.4	283			306500	±2900		
4.4	342			**348000**	**±4200**		
5.3	33	75280	±560				
5.3	38.2-39.1					81600	±460
5.3	102			93640	±310		
5.3	102.2-109	94760	±530				
5.3	111.5			107450	±610		
5.3	171.6	111840	±420				
5.3	188.7			136300	±1400		
5.3	225	190740	±790				
5.3	246			196300	±2100		
5.3	267	201800	±900				
5.3	318					283300	±2200
5.3	344			292900	±2000		
5.3	351	297700	±2800				
5.3	358					312300	±3200
5.3	374			319600	±2900		
6.5	30.1-30.8					33400	±100
6.5	118			**140800**	**±3200**		
6.5	139.5			**170800**	**±2600**		
6.5	152.5	**177700**	**±1000**				
6.5	165.7-166.4					193300	±1900
6.5	420			262700	±2000		
6.5	473			300500	±3800		
8	6.3					19850	±40
8	34					47020	±130
8	70.6			56550	±150		
8	104			**61650**	**±200**		
8	135	69240	±250				
8	266					137610	±500
8	306					165350	±690
8	337			189100	±1200		
8	28.2-34.8					23215	±50
8	37.2 - 38.9					32245	±85
8	430.5					159880	±690
8	634			273200	±2000		
9.5	19			144320	±580		
9.5	69					190000	±700
9.5	131			350000	±4700		
